# Best Bisphosphonate Threshold for 10-Year Vertebral and Non-vertebral Fracture Mitigation

**DOI:** 10.7759/cureus.59830

**Published:** 2024-05-07

**Authors:** Samer M Alboun, Eman Khreisat, Zaid E Alawneh, Khaled M Bani Hani, Rania F Khreisat, Mohammed A Al-Mughrabi, Bara’ah E Alshagoor, Rabaa I Alfarajat, Madher A Doumi, Mino Cycline

**Affiliations:** 1 Rehabilitation and Rheumatology, Jordanian Royal Medical Services, Amman, JOR; 2 Family Medicine, King Hussein Medical Center, Amman, JOR; 3 Internal Medicine, Jordanian Royal Medical Services, Amman, JOR; 4 Research and Development, Jordanian Royal Medical Services, Amman, JOR

**Keywords:** osteoporotic fracture, optimal threshold, nonvertebral fracture, vertebral fracture, bisphosphonate

## Abstract

Aims: This study was aimed to determine the ideal thresholds for bone mineral densities in our tested Jordanian cohort to initiate bisphosphonate pharmacotherapeutics in order to establish a national protocol for prescribing bisphosphonates that is tailored to the local population, rather than relying on global T and Z scores standards.

Methods: This retrospective study analyzed the entire population of adult patients at Prince Rashid bin Al-Hussein Hospital Rehabilitation and Rheumatology Center between August and October 2023 for the purpose of screening, monitoring, diagnosing, and treating osteoporosis. The study included 328 clients suspected to have osteoporosis, selected based on criteria such as primary osteoporosis or potential secondary osteoporosis. The study used two fracture risk assessment tools (FRAX) dichotomized states: <3% (negative state) and ≥3% (positive state), as well as <20% (negative state) and ≥20% (positive state). Binary logistic regression analysis, receiver-operating characteristic, and sensitivity analysis tests were performed sequentially to analyze the performance of prognosticators and sensitivity indices to evaluate their sensitivity, specificity, and accuracy indexes.

Results: The study involved 328 clients at a rehabilitation clinic, with 82.62% (271) females and 17.38% (57) males. The majority were aged between 60 and 69 years, with a slightly higher obesity rate in females. The study found that initiation of bisphosphonates in Jordanian cohorts with optimal bone mineral density thresholds of 0.775 g/cm^2^ may significantly reduce the risk of hip osteoporosis over 10 years, with sensitivity, specificity, and accuracy indexes of 78.6%, 88.46%, and 50.61%, respectively, with a performance utility of 0.896±0.026 (p-value<0.001), 95% CI (0.846-0.946).

Conclusion: Due to ethnicity differences, exploring regional or national specific bone mineral density thresholds for bisphosphonates initiation may be a better optional choice than adopting global T-score standards.

## Introduction

Osteoporosis causes disability, independence loss, hospitalizations, and a lower quality of life, affecting the healthcare system. Smoking, alcohol, inactivity, renal insufficiency, diabetes, vitamin D deficiency, hyperparathyroidism, and other factors are risk factors. Aging increases the risk of cardiovascular diseases, which are often found at OP diagnosis. Osteoporosis reduces bone mineral density (BMD), microarchitecture, strength, and fracture risk. Millions, mostly elderly, have osteoporosis. Degeneration of bone tissue microarchitecture, low bone mass, and weakness increase fracture risk. These fractures can cause chronic pain, disability, a lower quality of life, and higher mortality. By 2040, over 319 million people will be at risk of fragility fractures. Canadian hip fractures were 87 per 100,000 in 2015/16, rising to 1156 in 85-89-year-olds. These fractures can cause morbidity, decreased mobility, pain, quality of life, and mortality in five years [1-2].

Global estimates suggest one in three women and one in five men over 50 will fracture due to osteoporosis. Frail elderly people are more likely to develop osteoporosis. Fracturing can be devastating. Women are 40%-50% at risk of osteoporosis, while men are 13%-22%. The Jordanian health system lacks beds to treat the growing number of osteoporotic fracture patients. Health prevention must target these ages. There is insufficient international consensus on fragility fracture screening and treatment. Traditional screening detects osteoporosis by measuring BMD. Elderly patients' decreased BMD is linked to a general decline in functional ability, walking difficulties, increased fall risk, and higher fracture risk. Sedentary lifestyles also worsen cardiovascular issues, which lower bone quality and quantity [3-4].

Spinal osteoporosis affects biomechanically loaded vertebral trabecular bones. Due to bone weakness, vertebral fractures, the most common osteoporotic fracture, can occur with minimal trauma or daily activities. A trabecular bone score can assess bone quality in minimal trauma fracture patients with normal or osteopenic bone density. High scores indicate dense, well-connected bone microarchitecture. Conversely, a low TBS score indicates incomplete and weakened bone microarchitecture. However, the trabecular bone score is a cheap and easy indirect bone quality test. Age, BMI, and BMD were linked to a low trabecular bone score [5-6].

Early detection of osteoporosis fracture risk factors is essential for prevention, treatment, and cost reduction. Many clinical tools can predict osteoporotic fracture risk, but fracture risk assessment tools (FRAX) are the most popular. Recently, alternative methods for accurate and precise spinal osteoporosis fracture risk prediction have garnered attention. This study developed and validated a novel fracture risk-prediction model that estimates spinal osteoporosis patients’ osteoporotic fracture risk using demographic, clinical, biochemical, and radiological factors. Primary prevention screenings reduce fracture-related morbidity, mortality, and costs in patients without fragility fractures. Clinical risk factors estimate risk without BMD. Ageing and menopause-related bone loss cause primary osteoporosis in women. Medical conditions or medications can cause secondary osteoporosis [7-9].

A detailed clinical history, including drug history and exam, is needed for diagnosis. BMD, age, and previous fractures predict perimenopausal fractures. The 2008 computerized FRAX calculates each person’s 10-year hip and major osteoporotic fracture risk. Prior fragility fracture, smoking, parental hip fracture, excessive alcohol intake, systemic glucocorticoid use, rheumatoid arthritis (RA), and other secondary causes increase osteoporosis risk. A 10-year fracture probability independent of BMD is estimated by age, sex, BMI, and location. FRAX models cover 73 countries, with more planned. FRAX does not account for time since a previous fracture, which is important because risk is highest in the first 5 years and fluctuates. The FRAX algorithm uses T-Scores for femoral neck BMD instead of lumber spine BMD, which may misrepresent patient severity [10-13].

There is limited research in Mediterranean populations linking hip and lumbar bone mineral densities to elderly patients’ hip and vertebral osteoporotic fracture risk. Countries have varying treatment thresholds, but Canada, the USA, and others typically have a fixed 10-year major osteoporosis-related fracture probability of ≥20%. Bisphosphonates and denosumab prevent fragility fractures first. This study will determine the optimal threshold T-score for our Jordanian patients to start bisphosphonates.

## Materials and methods

This retrospective study examined the entire population of adult patients who visited our Prince Rashid bin Al-Hussein Hospital rehabilitation and rheumatology center between August and October 2023 for the purpose of screening, monitoring, diagnosing, and treating osteoporosis. The study has been approved by our Institutional Review Board (IRB) and is registered under the number 17_2/2024. The analysis was limited to clients who regularly attended and whose data was available for at least 80% of the specified time period, with a maximum of 20% of that time period being missing. Hakeem, our institution’s computer record system, dual-energy X-ray absorptiometry (DEXA) data files, and occasionally paper notes were utilized to get patient data, including demographics, anthropometrics, and DEXA-reported data. The DEXA scan revealed bone mineral densities in the femoral hip and lumbar, together with automatically calculated T and Z scores and an automatically determined 10-year probability of hip and major osteoporotic fractures.

This study comprised 328 clients who attended and were suspected to have osteoporosis. These clients were selected based on certain criteria, which included individuals over the age of 50 with primary osteoporosis or individuals under the age of 50 with potential secondary osteoporosis. Osteoporosis is defined by the World Health Organization as having a T-score of -2.5 or lower at the neck of the femoral or lumbar spine, as evaluated by DEXA. A T-score between -2.5 and -1 indicates osteopenia, while a T-score higher than -1 indicates normal bone health. The study took into account potential secondary factors that could contribute to osteoporosis, such as hyperparathyroidism, hypogonadism, RA, chronic kidney illness, cancer, or a previous spinal surgery. Patients who were not taking osteoporosis treatments, particularly antiresorptive agents such as bisphosphonates, were excluded.

The ages of the participants were classified into categories with a 10-year interval, ranging from the category of under 50 years to the category of 70 years or older. Similarly, BMIs were categorized in an ordinal manner, ranging from the underweight category (BMI<18.5 kg/m^2^) to the excessively obese category (BMI≥ 40 kg/m^2^). The FRAX scores were classified based on the established threshold percentages: 3% for hip fracture risk and 20% for significant osteoporotic fracture risk. The femoral hip and lumbar bone mineral densities of both patients were classified into two categories using appropriate cutoff values. The study findings indicated that the most effective thresholds for femoral hip and lumbar bone mineral densities in our Jordanian sample were determined to be 0.775 g/cm^2^ and 0.925 g/cm^2^, respectively. Using the identified optimal thresholds, we determined the ideal thresholds for bone mineral densities in our tested Jordanian cohort to initiate bisphosphonate pharmacotherapeutics. This was done in order to establish a national protocol for prescribing bisphosphonates that is tailored to the local population rather than relying on global T and Z score standards.

The study utilized two FRAX dichotomized states: FRAX<3% (negative state) and FRAX≥3% (positive state), as well as FRAX<20% (negative state) and FRAX≥20% (positive state). Indeed, the 10-year femoral hip osteoporotic fracture risk was based on the FRAX state of ≥3% vs. <3% and the 10-year overall major osteoporotic fracture risk was based on the FRAX state of ≥20% vs. <20%. Binary logistic regression analysis, receiver-operating characteristic (ROC), and sensitivity analysis tests were performed sequentially to assess the variability ranges, predictive accuracy, and percentage of cases that can be explained in the FRAX-related dependent variables based on participants’ bone mineral densities. In this study, we focused on analyzing the abstracted coefficients used to build binary regression models, the area under the ROC curve to measure the performance of prognosticators, and the sensitivity indices, including optimal thresholds, to evaluate the corresponding sensitivities, specificities, and accuracy indexes.

The evaluators analyzed the performances of the prognosticators by analyzing the area under the ROC curve, coupled with its standard error at the mean (AUROC ± SEM), within a 95% confidence interval (95% CI). A ROC area under the curve (AUROC) value greater than 0.70 was considered satisfactory, while a value over 0.80 was regarded as exceptional. The Delong approach was used to determine confidence intervals for the AUROC. However, the sensitivity indices that were included in this study were sensitivity (true positive rate or TPR), specificity (true negative rate or TNR), positive predictive value (PPV), negative predictive value (NPV), accuracy index (AI), and Youden’s index (YI).

Eligible tested patients were divided into two groups: the female cohort (Cohort I) and the male cohort (Cohort II). The nominal comparative variables were analyzed between the two cohorts using a Chi-square test to determine their distribution rates (at p-value< 0.05) and presented as numerical values (percentage). Associations’ strength was also characterized as odds ratios (OR). The Pearson chi-square statistic (χ2) is calculated by squaring the difference between observed and expected frequencies. The correlation between the tested variables in different gender-based cohorts was evaluated using interval-by-interval (Pearson, r) correlation and expressed as value ± standard error of value.

Microsoft Office LTSC Professional Plus 2021 Excel was used to collect and organize patients’ data. IBM SPSS Statistics version 25 was used for statistical analysis and summarizing the results of the study. This study used a significance level of 0.05.

## Results

The study included a cohort of 328 clients who attended our rehabilitation clinic between August 2023 and October 2023. Approximately 82.62% of the eligible tested clients were female (271), and about 17.38% were male (57). The ages of the tested patients were significantly distributed between the two genders’ cohorts, with a slight positive correlation toward the male cohort over the female cohort (+0.112±0.064, χ2=28.534, p-value<0.005). Most of the tested female patients’ ages were between 60 and 69 years old (99 (36.5%)), while the majority of tested male patients’ ages were 70 years or older (31 (54.4%)). In this study, the females’ cohort showed significantly higher obesity rates than the males’ cohort, with a slightly, significantly, and negatively significant correlation of -0.236±0.05, χ2=30.26, and a p-value of <0.005. The majority of the females’ cohort was categorized as obesity class I, followed by overweight. In contrast, most of the males’ cohort was classified as either normal weight or overweight, with a smaller percentage falling into obesity class I. Among the evaluated individuals, 38 women (14.0%) and four men (7.0%) were identified as having a greater 10-year risk of hip osteoporotic fracture, based on FRAX scores of ≥3% for higher risk and <3% for lower risk. The Pearson correlation for the higher 10-year risk of hip osteoporotic fracture in the tested men compared to the tested women was negatively insignificant (-0.079±0.045, x2=2.070, p-value=0.150), although we revealed in this study a positively significant Pearson correlation for the higher femoral hip BMD in the male cohort compared to the female cohort (+0.130±0.042, x2=5.529, p-value=0.019) with an OR of 3.021 (95% CI; 1.156-7.897). The risk estimates for the tested men compared to the tested women in developing hip osteoporotic fracture during a 10-year period were statistically negligible, with a value of 0.463 (95% CI; 0.158-1.353) and a distribution rate of around 7% (4 males) versus approximately 14% (38 females). The comparison results for the evaluated variables across the two gender-based cohorts (Cohorts I and II) are fully presented in Table 1.

**Table 1:** Comparatively studied independent variables across the dichotomized gender-based cohorts; I-II. Data results of the comparative variables between the two tested cohorts were statistically analyzed by the Chi-square test (at p-value< 0.05) and expressed as Numbers (Percentage). The strength of associations or risk estimates was also described as OR. The Pearson chi-square statistic (χ2) which involves the squared difference between the observed and the expected frequencies was also expressed. The interval-by-interval (Pearson, R) correlations were expressed as value ± standard error of value and the star donation was referred to the statistical significance. The studied patients in this study were dichotomously categorized into two comparative cohorts; the females’ cohort (Cohort I) and the males’ cohort (Cohort II). The optimal thresholds of 0.775 g/cm2 and 0.925 g/cm2 for bone mineral densities of both femoral neck and lumbar, were adopted based on running an ROC test sequentially with binary logistic regression and sensitivity analysis, to abstract these thresholds that were consequently used in this study to dichotomize the categorical tested independent variables; fH_BMD and L_BMD. The tested FRAX variable, used both the 3% and 20% for referring to the 10-year risk of hip osteoporotic fracture and major osteoporotic fracture, respectively. *Significant (p<0.05) fH_BMD: Femoral hip bone mineral density in g per cm^2^. FRAX: Fracture risk assessment tool. LBMD: Lumbar bone mineral; density in g/cm^2^. BMI: Body mass index in kg/m^2^.

	Female (271, 82.62%)	Male (57, 17.38%)	Total (328, 100%)	OR	R	χ^2^	p-value
Age (Years)							
<50	32 (11.8%)	10 (17.5%)	42 (12.8%)	NA	+0.112±0.064*	28.534	0.000
50-59	76 (28.0%)	10 (17.5%)	86 (26.2%)				
60-69	99 (36.5%)	6 (10.5%)	105 (32.0%)				
>=70	64 (23.6%)	31 (54.4%)	95 (29.0%)				
fH_BMD (g/cm^2^)							
<0.775	61 (22.5%)	5 (8.8%)	66 (20.1%)	3.021 (95% CI; 1.156-7.897)	+0.130±0.042*	5.529	0.019
≥0.775	210 (77.5%)	52 (91.2%)	262 (79.9%)				
FRAX							
<3%	233 (86.0%)	53 (93.0%)	286 (87.2%)	0.463 (95% CI; 0.158-1.353)	-0.079±0.045	2.070	0.150
≥3%	38 (14.0%)	4 (7.0%)	42 (12.8%)				
FRAX							
<20%	265 (97.8%)	57 (100.0%)	322 (98.2%)	NA	-0.063±0.013	1.286	0.257
≥20%	6 (2.2%)	0 (0.0%)	6 (1.8%)				
LBMD (g/cm^2^)							
<0.925	167) 61.6%)	26 (45.6%)	193 (58.8%)	1.915 (95% Ci; 1.076-3.405)	+0.123±0.056*	4.984	0.026
≥0.925	104 (38.4%)	31(54.4%)	135 (41.2%)				
BMI (kg/m^2^)							
Underweight	1 (0.4%)	0 (0.0%)	1 (0.3%)	NA	-0.236±0.054*	30.26	0.000
Normal weight	18 (6.6%)	17 (29.8%)	35 (10.7%)				
Overweight	73 (26.9%)	17 (29.8%)	90 (27.4%)				
Obese I	84 (31.0%)	12 (21.1%)	96 (29.3%)				
Obese II	60 (22.1%)	9 (15.8%)	69 (21.0%)				
Obese III	35 (12.9%)	2 (3.5%)	37 (11.3%)				

**Figure 1:**
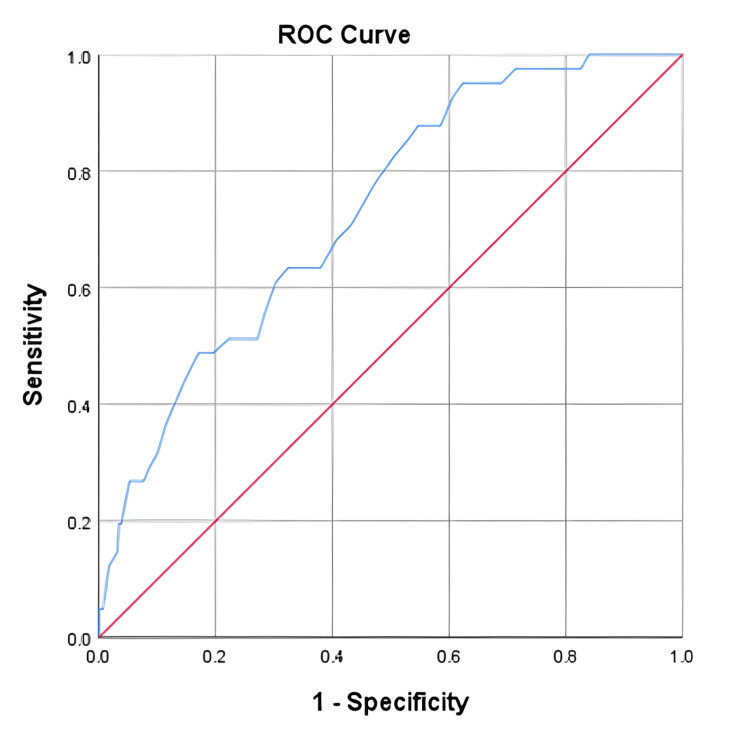
Receiver-operating characteristic (ROC) analysis was conducted to illustrate sensitivity vs false positive values for the tested lumbar bone mineral density (LBMD) against the probability for FRAX≥3% in our Jordanian participants. We examined the AUROC±SEM, which was found to be 0.732±0.039 (95% CI; 0.656-0.808). The study determined that the optimal performance of the participants’ LBMD was achieved at a threshold value of 0.925 g/cm^2^, as indicated by Youden's index rate of 33.1%. In the case of the investigated FRAX versus LBMD, 41 cases were classified as having a positive actual status (FRAX≥3%) and 287 cases were classified as having a negative actual state (FRAX<3%). A lower LBMD value indicates more compelling evidence for a positive current condition. In addition to the optimal fH_BMD for our Jordanian-tested participants, the conducted sensitivity analysis revealed that the accompanied sensitivity indices of sensitivity, specificity, positive and negative predictive values, and AI were 87.8%, 45.3%, 18.65%, 96.30%, 50.61%, respectively.

**Figure 2:**
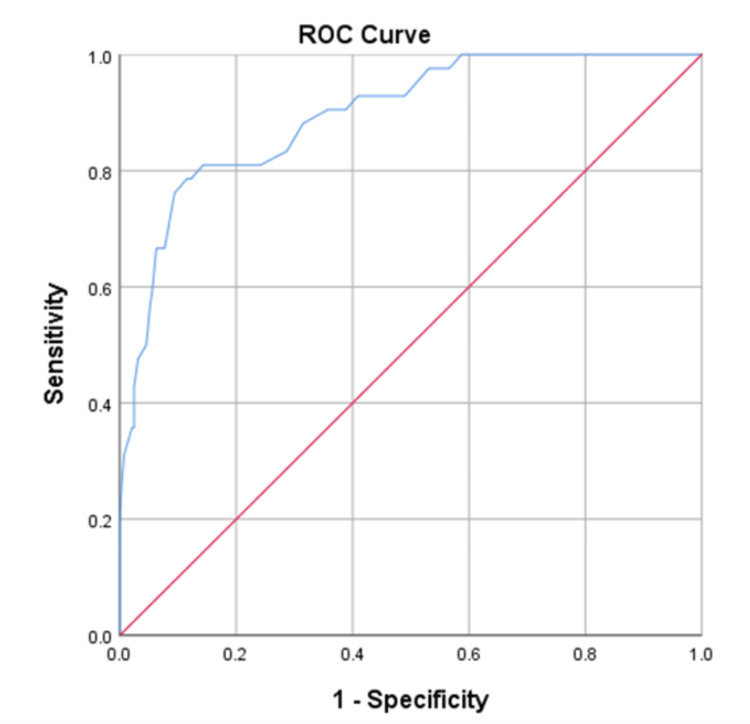
Receiver-operating characteristic (ROC) analysis was conducted to illustrate sensitivity vs false positive values for the tested femoral hip bone mineral density against the probability for FRAX≥3% in our Jordanian participants. We examined the AUROC±SEM, which was found to be 0.896±0.026 (95% CI; 0.846-0.946). The study determined that the optimal performance of the participants’ femoral hip bone mineral density (fH_BMD) was achieved at a threshold value of 0.775 g/cm^2^, as indicated by Youden's index rate of 67.03%. In the case of the investigated FRAX versus fH_BMD, 42 cases were classified as having a positive actual status (FRAX≥3%) and 286 cases were classified as having a negative actual state (FRAX<3%). A lower fH_BMD value indicates more compelling evidence for a positive current condition. In addition to the optimal fH_BMD for our Jordanian-tested participants, the conducted sensitivity analysis revealed that the accompanied sensitivity indices of sensitivity, specificity, positive and negative predictive values, and AI were 78.6%, 88.46%, 50%, 96.56%, 77.14%, and 67.03%, respectively.

The ROC analyses were conducted to illustrate the sensitivity vs. false positive values for both femoral and hip bone mineral densities against the probability of a FRAX binary state. Accordingly, both the area under the ROC curves and the sensitivity indices were obtained. The AUROCs±SEMs for the tested femoral and lumbar hip bone mineral densities against the probability for (FRAX≥3% over FRAX<3%) and (FRAX≥ 20% over FRAX<20%) were determined at 0.896±0.026 (p-value<0.001), 95% CI (0.846-0.946), and 0.732±0.039 (<0.001), 95% CI (0.656-0.808), respectively. Indeed, 42-case and 41-case were processed as positive states (FRAX≥3% and FRAX≥20%), and 286-case and 287-case were processed as negative states (FRAX<3% and FRAX<20%), respectively. Sensitivity analysis revealed optimal femoral hip and lumbar BMD thresholds of 0.775 g/cm2 and 0.925 g/cm2 for initiation bisphosphonates’ pharmacotherapies in our studied Jordanian cohort, with yielded sensitivities, specificities, and AIs of 78.6% vs. 87.8%, 88.46% vs. 45.3%, and 77.14% vs. 50.61%, respectively. The ROC analyses’ results, accompanied by the corresponding sensitivity indices’ outcomes, including both positive and negative predictive values and Youdens’ indexes, were comprehensively detailed in Figures 1-2.

**Figure 3:**
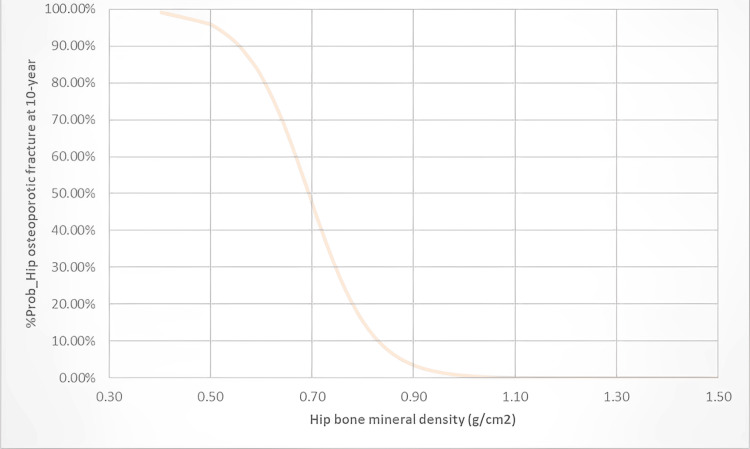
The binary logistic illustration for the participants' femoral hip bone mineral density (fH_BMD) against the probability of their FRAX being >=3%. A binary logistic regression model was created to examine the relationship between the participants’ femoral hip bone mineral density (fH_BMD) and the probability of their FRAX being ≥3% over <3%. The model was constructed as [e (11.101-16.008×fH_BMD)/1+e (11.101-16.008×fH_BMD)]. At fHBMD value of 0.775 g/cm^2^, the probability of a patient having FRAX ≥3% was determined to be 21.33%. The logistic-based model demonstrated statistical significance, with a chi-square value of 12.026 and a p-value of less than 0.0005. The variance accounted for in the dependent variable by our model ranges from 25.9% to 48.3% depending on whether you use the Cox & Snell R2 or Nagelkerke R2 methods, respectively. Additionally, our model accurately classified 90.5% of the cases. Y-Axis refers to the %probability for the 10-year risk of hip osteoporotic fracture. X-Axis refers to the participants' femoral hip bone mineral density in g per cm^2^

**Figure 4:**
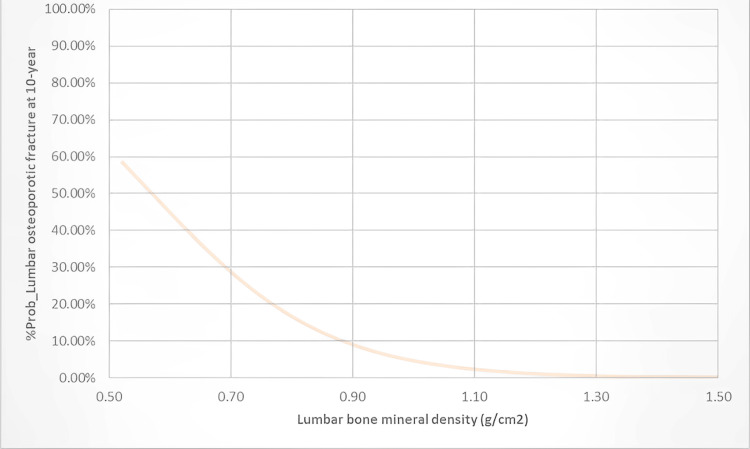
The binary logistic regression illustration for the participants' lumbar bone mineral density (LBMD) against the probability of their FRAX being >=20%. A binary logistic regression model was created to examine the relationship between the participants’ LBMD and the probability of their FRAX being ≥20% over <20%. The model was constructed as [e (3.994-6.995×LBMD)/1+e (3.994-6.995×LBMD)]. At LBMD value of 0.925 g/cm^2^, the probability of a patient having FRAX ≥20% was determined to be 7.75%. The logistic-based model demonstrated statistical significance, with a chi-square value of 8.970 and a p-value of less than 0.05. The variance accounted for in the dependent variable by our model ranges from 8.2% to 15.5% depending on whether you use the Cox & Snell R2 or Nagelkerke R2 methods, respectively. Additionally, our model accurately classified 88.1% of the cases. Y-Axis refers to the %probability of the 10-year risk of the participants' vertebral and non-vertebral major osteoporotic fracture. X-Axis refers to the participants' LBMD in g per cm^2^

A binary logistic regression analysis revealed that both the femoral hip and lumbar bone mineral densities were significantly correlated with the FRAX-based binary dependent variables of investigation. However, the variability ranges on the probability of FRAX≥ 3% or FRAX≥20%, and the percentage cases that can be explained by the femoral hip or lumbar bone mineral densities were 25.9%-48.3%, 90.5% (or 8.2%-15.5%, 88.1%), respectively. The binary logistic regression model for the probability of FRAX≥3% or FRAX≥20% was constructed as [e (11.101-16.008×fH_BMD)/1+e (11.101-16.008×fH_BMD)] or [e (3.994-16.995×LBMD)/1+e (3.994-6.995×LBMD)], respectively. The probability of a 10-year risk of hip osteoporotic fracture at the explored optimal femoral hip BMD of 0.775 g/m^2^ in our studied Jordanian cohort was determined at 21.33%. While the probability of a 10-year risk of major osteoporotic fracture at the explored optimal lumbar BMD of 0.925 g/m^2^ in our studied Jordanian cohort was determined at 7.75%. The binary logistic regression illustrations with their details are thoroughly presented in Figures 3-4.

## Discussion

Osteoporosis is a disease characterized by low bone mass and structural deterioration of bones, leading to bone fragility and increased susceptibility to fractures of the hip, spine, and wrist. It is a significant public health concern in an aging population and the prevalence of osteoporosis will continue to rise as the population continues to age. The increase in mortality after a fracture is another aspect of the disease severity. White women have the greatest risk of experiencing an osteoporotic fracture, with vertebral or hip fractures carrying an increase in mortality estimated at 20%, higher than the mortality for the same age and sex without a hip fracture. BMD testing is closely related to the occurrence of osteoporotic fractures and several health administrations recommend testing for anyone with a break in a bone occurring with minimal trauma, those with X-ray evidence of a vertebral fracture, and anyone with back pain suggesting a fracture [14-15].

The FRAX, developed by WHO, evaluates the 10-year probability of a hip or major osteoporotic fracture in a patient based on individual patient models and femoral neck BMD. It determines the threshold for intervention by determining the efficacy of treating or not treating the fracture. The tool considers multiple clinical risk factors associated with fracture probability and BMD at the femoral neck. It determines whether to initiate treatment solely based on age and sex or whether BMD testing is cost-effective in determining an intervention. By determining the probability of a patient sustaining a hip or major osteoporotic fracture in the next 10 years, it identifies patients potentially in need of bisphosphonate treatment [16-17].

Indeed, in a study by Dell et al., the initiation of bisphosphonate treatment after an osteoporotic fracture is measured in correspondence to fracture probability FRAX and BMD at the femoral neck. A high fracture rate is considered the occurrence of another osteoporotic fracture post-initial one, during the minimum three-year follow-up duration. Measurement of fracture risk reduction using bisphosphonate treatment is determined via considering individuals free from any new fractures during follow-up, compared to those who have new fractures after the initial one, ultimately with the use of Cox regression analysis. The optimal threshold for bisphosphonate initiation is often based on a BMD-related T-score of less than -2.5 in postmenopausal women or a previous fragility fracture. However, a recent systematic review by Schousboe et al. analyzed the evidence for BMD thresholds at which postmenopausal women and men should begin taking drugs to prevent fractures, concluding that drug treatment to prevent fractures is generally indicated in postmenopausal women when the 10-year probability of a hip fracture exceeds 3% or of a major osteoporotic fracture exceeds 20-25%, based on country-specific thresholds [17-19].

A recent study found that postmenopausal women with a fracture and low trauma BMD at the hip have a higher probability of a further forearm fracture than those with normal BMD. Therefore, it could be recommended that a postmenopausal woman with a fracture and low trauma BMD at the hip should be offered treatment with bisphosphonate. In the current healthcare climate, there is mounting pressure for clinicians to rationalize their treatment decisions, and the decision to initiate bisphosphonate treatment is highly dependent on the attended clients. Factors such as previous fractures, steroid usage, and other disease-specific factors will likely influence the decision, but the BMDs result is likely the primary factor [20-21].

Yu and Finkelstein’s study revealed that bone density testing rates have plateaued, with only 52% of eligible women having a test over a 7-year period, despite US guidelines for universal screening of postmenopausal women aged 65 and older. This discrepancy is concerning, as half of the US postmenopausal women will experience an osteoporotic fracture in their lifetime [22]. Celi et al.’s study concluded that bone densitometry measurements, such as computed tomography, ultrasound, and DEXA, offer advantages like interpreting BMD results, predicting fracture risk, targeting anti-fracture therapies, and monitoring treatment response. However, recent questions about the objective limits of DEXA have raised doubts about its clinical effectiveness [23]. In Schreiber et al.’s study, clinical computed tomography scans can provide an alternative method for determining regional BMD, potentially aiding in fracture risk assessment, osteoporosis diagnosis, and early treatment initiation [24]. Gruenewald et al.’s study revealed that volumetric BMD assessment accurately predicts the 2-year risk of osteoporosis-associated fractures in at-risk patients in which lower volume values are associated with increased fragility fracture risk with an optimal volume threshold of 93.70 mg/cm^3^ and corresponding 85.45% sensitivity and 89.19% specificity for predicting fractures within 2 years [25].

In our study, we adopted the FRAX as a dichotomized states in the conducted statistical tests. The binary logistic regression, ROC, and sensitivity tests analyzed the participants’ BMD against these FRAX-based binary states to yield the outcomes’ results of interest; variation ranges, quality of prediction, performances utilities’ area under curve, binary constructed models, and sensitivity indices including optimal threshold for initiation bisphosphonates’ drugs. This study is unique in that it evaluated the participants’ BMD at the time of pre-fracture. It determined the binary logistic regression relationships between participants’ femoral hip or lumbar BMDs and FRAX-based binary states, which were significant with variability ranges and % cases probability prediction of (25.9%-48.3%, 90.5%) or (8.2%-15.5%, 88.1%), respectively. It explored the corresponding femoral hip and lumbar bone mineral densities’ optimal thresholds which were determined at 0.775 g/cm^2^ and 0.925 g/cm^2^ for initiation bisphosphonates’ pharmacotherapies in our studied Jordanian cohort, with yielded sensitivities, specificities, and AIs of 78.6% vs. 87.8%, 88.46% vs. 45.3%, and 77.14% vs. 50.61%, respectively. It also investigated the associated performance utilities which were determined at 0.896±0.026 (p-value<0.001), 95% CI (0.846-0.946), and 0.732±0.039 (<0.001), 95% CI (0.656-0.808), respectively.

This study has several drawbacks. The retrospective single-center observational design limits its usefulness in adopting these regional-specific yielded BMDs thresholds as a surrogate plan for initiation bisphosphonates pharmacotherapeutics. The relatively small sample design and depending a binary state of FRAX as an investigated state of interest instead of conducting biochemical or radiological derived depending variable of investigation. However, this study may be of valuable in finding an assessment way for determining the eligibility for initiation bisphosphonates medications for attended clients in a regional, national specific manner rather than global fixed standards matter.

## Conclusions

In summary, we concluded that adopting regional individualized specific femoral hip and lumbar bone mineral densities may be more appropriate for prioritizing bisphosphonate medication due to ethnicity differences. In this study, we revealed that optimal femoral hip and lumbar bone mineral densities of 0.775 g/cm^2^ and 0.925 g/cm^2^, respectively, may be more appropriate for initiation bisphosphonates’ pharmacotherapies in our tested Jordanian attended clients.
